# Alteration of pulmonary function in diabetic nephropathy

**DOI:** 10.1186/2251-6581-12-15

**Published:** 2013-04-24

**Authors:** Gita Shafiee, Mohammad E Khamseh, Nader Rezaei, Rokhsareh Aghili, Mojtaba Malek

**Affiliations:** 1 Endocrinology and Metabolism Research Center, Endocrinology and Metabolism, Research Institute, Tehran University of Medical Sciences, Tehran, Iran; 2 Endocrine Research Center (Firouzgar), Institute of Endocrinology and Metabolism, Tehran University of Medical Sciences, Karimkhan St, Tehran 1593748711, Iran; 3 Department of Internal Medicine, Firouzgar Hospital, Tehran University of Medical Sciences, Tehran, Iran

**Keywords:** Pulmonary function, Type 2 diabetes, Diabetic nephropathy

## Abstract

**Background:**

Type 2 diabetes mellitus is increasing worldwide with an alarming rate. It is associated with the development of various chronic complications. The aim of this study was to explore the alteration of pulmonary function, and its association with renal complications in people with type 2 diabetes mellitus.

**Methods:**

This cross-sectional study was conducted on three groups; 40 diabetic subjects without nephropathy (urinary albumin<30 mg/day), 40 subjects with nephropathy (urinary albumin≥30 mg/day), and 40 healthy subjects as the control group. The subjects with nephropathy were divided into those with microalbuminuria (urinary albumin=30-300 mg/day) and those with macroalbuminuria (urinary albumin>300 mg/day) .Diabetic subjects were matched to the control group in terms of age, sex, and BMI. Pulmonary function tests were performed and the results were compared between groups.

**Results:**

Forced vital capacity (FVC; % predicted), forced expiratory volume in 1 second (FEV1; % predicted), and peak expiratory flow (PEF; % predicted) were significantly lower in subjects with diabetic nephropathy compared to the healthy controls (P<0.05). Meanwhile, in diabetic subjects, FVC and FEV1 were lower in those with diabetic nephropathy compared to those with normal albumin excretion (P<0.05). On the other hand, FEV1/FVC was significantly higher in diabetic people with nephropathy.

Furthermore, a significant difference was observed between FVC and FEV1 in diabetic people with microalbuminuria compared to those with macroalbuminuria.

**Conclusions:**

This study showed that the pulmonary function was impaired in people with Diabetes. The progression of diabetic nephropathy to more advanced stages was also associated with more impairment of pulmonary function.

## Introduction

Type 2 diabetes mellitus is associated with the development of micro- and macrovascular complications [1]. The development of these complications can be explained by the biochemical adjustment of connective tissue as well as by microangiopathy due to protein glycosylation induced by chronic hyperglycemia [2,3].

The pulmonary alveolar- capillary network represents the largest microvascular structure in the body that could be potentially affected by diabetic microangiopathy [4]. Some studies showed that in diabetic subjects, loss of elastic recoil secondary to collagen and elastin changes [5], chronic inflammation [6], autonomic neuropathy involving pulmonary muscles, as well as microangiopathy of the alveolar capillaries [1] can cause pulmonary dysfunction. However, pulmonary complications may be under- diagnosed clinically [4]. It has also been demonstrated that the pulmonary and other late complications of diabetes share a similar microangiopathic background [2,3].

An association between diabetic nephropathy and alteration of lung function had been described in type 1 diabetes [2]. However, in type 2 diabetes, limited studies have been done to explore the alteration of pulmonary function in the presence of diabetic nephropathy.

The aim of this study was to explore the effect of diabetic nephropathy on pulmonary function in people with type 2 diabetes.

## Materials and methods

This cross- sectional study was carried out from February to Jul 2011 to assess pulmonary function in people with type 2 diabetes compared with healthy individuals.

A total of 120 people were enrolled in the study using consecutive sampling methods and comparison was done among three groups, IE, diabetic people without nephropathy, diabetic people with nephropathy, and healthy controls. Nephropathy was defined as presence of ≥30 mg albumin in a 24 hour urine sample collection, excluding a falsely elevated urinary albumin excretion.

Those with history of cardiopulmonary, connective tissue, overt renal disorders and end stage renal disease (GFR < 60), patients with HbA1C>10% and Body mass index ≥ 35 Kg/m^2^ were excluded as well as those with a history of cigarette smoking at any time. All subjects included did not have a history of chronic cough, sputum production, dyspnea or any other systemic disorder that may interfere with the results. The urinary albumin was measured in the absence of urinary tract infection. The patients with nephropathy were divided into those with microalbuminuria (urinary albumin=30-300 mg/day) and those with macroalbuminuria (urinary albumin>300 mg/day). The serum levels of serotonin were within the normal range of all groups. Diabetic subjects were matched to the control group in terms of age, sex, and BMI.

Weight was measured, while they were barefoot with minimal clothing using digital scales and was recorded to the nearest 100 g. Height was measured in a standing position, without shoes, using a tape meter with shoulders in normal alignment. Body mass index (BMI) was calculated as weight in kilograms divided by height in meters squared. To avoid interobserver error, all measurements were taken by one person. Blood samples were taken in a sitting position according to the standard protocol and centrifuged within 30-45 min of collection. The analysis of samples was performed using the selectra 2 auto-analyzer (Vital scientific, Spankeren, Netherlands). Glycosylated hemoglobin (HbA1C) was measured using ion exchange chromatography (DS5 Analyzer, Drew Scientific Limited, Cumbria, U.K). The urinary albumin concentration was measured by nephelometric method.

Pulmonary function tests were performed in the morning between 9:00-11:00 AM in a sitting position after a resting period, using a standard spirometer (ML3500 MK8 MicroLab Spirometer U.K). Spirometry was performed by trained and certified pulmonary technicians in accordance with the American Thoracic Society Guidelines [7]. Measured parameters were forced vital capacity (FVC), forced expiratory volume in 1 second (FEV1), vital capacity (VC) and peak expiratory flow (PEF). The highest value for each volume from three technically acceptable maneuvers were used for evaluation [8].

The values expressed as percentage of the predicted normal value [9] were calculated for these parameters.

Informed consent was obtained from those eligible subjects who desired to participate in the study. Ethical approval was granted from research Ethics' committee of Institute of Endocrinology and Metabolism, Tehran University of Medical Sciences.

### Statistical analysis

All data are shown as mean ± SD unless otherwise stated. Statistical evaluation was performed by analysis of variance for continuous and chi- square test for categorical variables. The statistical analysis of the percent predicted values of respiratory parameters tests was used for the explanation of the results. Differences in pulmonary function parameters between patients with and without albuminuria were examined using multivariable linear regression, adjusting for age and sex. To identify the variables most strongly associated with spirometric measures, a linear regression model was fitted considering age, gender, HbA1C, duration of diabetes and albuminuria as independent variables.

SPSS for Windows (version 16; SPSS Inc., Chicago, ILL) was used for data analyses and P-values <0.05 were considered as statistically significant.

## Results

Forty diabetic people without albuminuria (62.5% female), 40 diabetic people with albuminuria (60% female), and 40 healthy individuals (42.5% female) were enrolled in this study. Among those with albuminuria, 36.2% had microalbuminuria and 13.8% had macroalbuminuria. The mean age of the participants was 53.6±11.9 years, and the mean for BMI was 28.8±4.1 kg/m^2^. Table 1 shows the baseline characteristics of the study population. Diabetic people with normoalbuminuria were slightly younger, and had a shorter duration of diabetes as compared with diabetic people with albuminuria, but there was no statistically significant difference in baseline characteristics of the participants.

**Table 1:** Baseline characteristics of the study population

	**Diabetics**		**Non diabetics**	P value*
	**Normoalbuminuria (n=40)**	**Albuminuria (n=40)**	**(n = 40)**	
Male (%)	37.5	40	47.5	NS
Age (years)	52.0±11.5	55.2±12.3	51.9±7.2	NS
Height (cm)	159.7±9.7	158.5±8.1	160.1±8.7	NS
Weight(kg)	72.7±12.9	73.0±10.7	74.2±13.4	NS
Body Mass Index (kg/m2)	28.5±4.0	29.1±4.2	27.4±3.6	NS
Duration of diabetes (years)	8.9±5.7	10.8±7.2	---------	
HbA1C (%)	8.4±2.0	8.4±1.9	----------	
Albumin excretion rate (mg/24 h)	18.8±1.2	334.4±88.9	----------	

Data are means (SD) unless indicated otherwise. NS: Non Significant.

*P value (Non diabetics vs. all diabetic patients).

Pulmonary function parameters of the type 2 diabetic people and healthy individuals are shown in Table 2. Comparing with the healthy controls, significant reduction of FVC (% predicted), FEV1 (% predicted), and PEF (% predicted) were found in diabetic people with nephropathy (P<0.05). FVC (% predicted P<0.001), and FEV1 (% predicted P<0.05) were lower in diabetic people with nephropathy compared to those with normal renal albumin excretion. On the other hand, FEV1/FVC (% predicted P<0.05) was significantly higher in subjects with nephropathy than these without nephropathy. After adjustment for sex and age the FVC, FEV1 and PEF remained significantly lower in people with nephropathy (P<0.05).

**Table 2:** Pulmonary function parameters of diabetic people and healthy individuals

	**Diabetic**		**Nondiabetics**
	**Normoalbuminuria**	**Albuminuria**	**(n = 40)**
	**(n=40)**	**(n=40)**	
FVC (predicted %)	115.45(16.44) ^c^	99.72(12.34) ^a, c^	111.35(14.87)
FEV1 (predicted %)	112.67(16.24)^b^	101.47(13.58)^a, b^	109.82(15.33)
PEF (predicted %)	90.95(17.37)	83.70(19.05)^a^	96.82(16.11)
FEV1/FVC (predicted %)	102.55(7.26)^b^	106.52(8.07)^b^	103.47(6.59)

Data are mean(SD), FVC: Forced Vital Capacity, FEV1: Forced Expiratory Volume in 1s, PEF: Peak Expiratory Flow.

Diabetic people versus healthy individuals: ^a ^P<0.05.

Diabetic people with no albuminuria versus those with albuminuria: ^b ^P<0.05; ^c^ P<0.001.

Albuminuria was associated with alteration of all pulmonary function parameters (FVC, FEV, FEV1/FVC), except PEF, independent of other risk factors (P<0.05) (Table 3).

**Table 3:** The effect of albuminuria on pulmonary function parameters in diabetic people after adjusting other risk factors*

	***β***	**S.E**	**P value**
FVC (predicted %)	-0.51	4.10	<0.001
FEV1 (predicted %)	-0.37	3.91	0.006
PEF (predicted %)	-0.22	4.76	0.11
FEV1/FVC (predicted %)	0.29	1.97	0.02

* linear regression model after adjusting for age, sex, Glycosylated hemoglobin (HbA1C), and duration of diabetes.

FVC: Forced Vital Capacity, FEV1: Forced Expiratory Volume in 1s, PEF: Peak Expiratory Flow.

**Figure 1:**
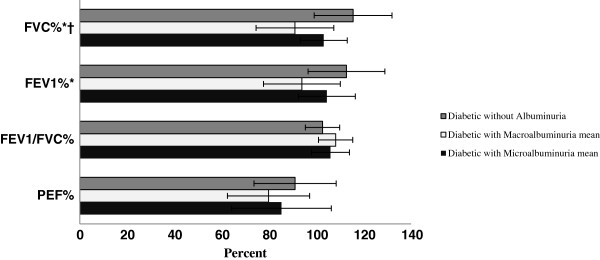
**Pulmonary function parameters according to albuminuria in diabetic people. **FVC: Forced Vital Capacity, FEV1: Forced Expiratory Volume in 1s, PEF: Peak Expiratory Flow. * P<0.05, Diabetic people with no albuminuria versus those with albominuria (micro and macroalbuminuria). † P<0.05, Diabetic people with macroalbuminuria vs. those with microalbuminuria.

Figure 1 compares the differences of pulmonary function parameters according to albuminuria in diabetic people. As the figure illustrates, the mean percent predicted values were 90.91% ± 14.21 vs. 103.07% ± 9.90, P<0.05 for FVC, and 93.82 ± 15.02 vs. 104.38 ± 12.02, P=0.05 for FEV1, in patients with macroalbuminuria and with microalbuminuria, respectively.

## Discussion

The results of the present study indicate that the pulmonary dysfunction is more pronounced in subjects with diabetic nephropathy, independent of other risk factors. Moreover, the progression of nephropathy to the more advanced stages was also associated with more impairment of pulmonary function.

Our results are generally consistent with prior studies, which demonstrated lower FVC and FEV1 in adults with diabetes as compared to non-diabetic counterparts [10,11].

People with diabetes are at increased risk of microvascular complications. The main reasons of these complications are connective tissue changes as a result of microangiopathy due to glucosylation of proteins induced by chronic hyperglycemia [1]. Recently in a systematic review, Klein et al showed that lung is a large microvascular organ and may be affected by microangiopathy in diabetic people [12]. Adults with diabetes have decreased lung function compared with adults without diabetes, regardless of other risk factors. The most important risk factors are glycemic control and diabetes duration. However, along with the presence of external risk factors, some associations have also been noted considering diabetes complications [10,13].

The Diabetes Control and Complications Trial (DCCT) demonstrated that there was a strong relationship between diabetic retinopathy and elevated albumin excretion [14]. The EURODLAB study showed that diabetic retinopathy, in association with increased blood pressure, is an important independent risk factor for diabetic nephropathy progression [15].

In addition, it has been demonstrated that the pulmonary and other organ systems of people with diabetes share a similar microangiopathic background. There is strong epidemiological evidence that peak expiratory flow rate is decreased in diabetic people with microvascular complications compared to those without microvascular complications [16].

Chronic complications of diabetes have relatively similar frequency and severity which might be due to an identical mechanism. Postmortem studies of diabetic people showed an increased thickness of the alveolar wall and small vessel. These changes might be responsible for the limitation of lung expansion and reduction in the ventilatory capacity. In the kidney, thickness of glomerular capillaries causes impaired selectivity for proteins and an increase in albumin excretion rate. All these support the importance of an etio-pathologic mechanism in the development of diabetic microvascular complications [11,17].

In previous studies, a marked alteration of the pulmonary function was found in type 1 diabetic patients with end- stage renal disease, suggesting a high prevalence of restrictive pulmonary disease in the presence of diabetic nephropathy [9,18], To our knowledge there are a few studies about the effect of diabetic nephropathy in its earlier stages on pulmonary function especially in type 2 diabetic people. In the current study, the significant reduction of pulmonary function parameters was more pronounced in patients with albuminuria comparing to those with normal urinary albumin excretion.

Some clinical studies have found that pulmonary function parameters in people with diabetes are inversely correlated with glycemic control, duration of diabetes, and its severity. Some other risk factors including age, sex, and genetic factors, might be involved as well [17]. In the Framantle Diabetes Study [10], diabetes duration was more influential than the state of glycemic control. Obesity and vascular disease were independently and inversely associated with spirometric parameters. In our study, presence of diabetic nephropathy was associated with reduced pulmonary function parameters whereas other risk factors such as duration of diabetes and HbA1C were not. Although a relationship between glycemia and impaired lung function could be present in diabetic patients, our data provide some evidence that existing microvascular complication may be more important than other pulmonary risk factors. Although suggested mechanisms for impaired pulmonary function in diabetic patients include glycosylation of proteins, the lack of association of pulmonary parameters with glycemic control and duration of diabetes in this study, shows a more complex model of diabetic complications- related pulmonary damage. Among the main mechanisms, systematic and local inflammation [6], autonomic neuropathy involving the respiratory muscles [19], and hypoxia- induced insulin resistance could be responsible for impaired pulmonary functions [20]. Left ventricular diastolic dysfunction observed in diabetic people with microalbuminuria, and central volume expansion may provide pulmonary impairment in patient with albuminuria [21]. As diabetic complications are interrelated, early detection of microvascular disease in its earliest stages might be beneficial [22].

There are some limitations that should be considered when examining the results of this study. The first limitation is the use of a cross- sectional design to find the pulmonary impairment in diabetic patients with albuminuria. Secondly, the sample size was fairly small and limited the power to recognize small differences between groups. The other limitation was excluding patients with cardiopulmonary disorders just based on history taking.

Our findings showed that the association between albuminuria and reduced pulmonary function was not explained by concurrent respiratory infections or heart failure. We also showed that progression of diabetic nephropathy was associated with more deterioration of pulmonary function.

## Conclusion

Our findings showed that the pulmonary function was impaired in people with diabetes. The severity of pulmonary dysfunction seems to be correlated with the severity and stage of diabetic nephropathy. Longitudinal studies are needed to examine pulmonary function in diabetic people as a marker of microvascular involvement in diabetes.

## Competing interests

The authors declare that they have no competing interests.

## Authors’ contribution

GS participated in the drafting of the manuscript, MM and NR participated in the study design, RA critically revised the manuscript, and MEK was main supporter of the study and contributed in the writing of specific sections of the manuscript. All authors read and approved the final manuscript.

## References

[B1] Klein OL Krishnan JA Glick S Smith LJ Systematic review of the association between lung function and Type 2 diabetes mellitus Diabet Med 2010 27 977 987 20722670 10.1111/j.1464-5491.2010.03073.x

[B2] Orasanu G Plutzky J The pathologic continuum of diabetic vascular disease J Am Coll Cardiol 2009 53 5 Suppl S35 S42 19179216 10.1016/j.jacc.2008.09.055 PMC2663393

[B3] Aronson D Hyperglycemia and the pathobiology of diabetic complications Adv Cardiol 2008 45 1 16 18230953 10.1159/000115118

[B4] Hsia CC Raskin P Lung involvement in diabetes: does it matter? Diabetes Care 2008 31 828 829 10.2337/dc08-0103 18375433

[B5] Hamlin CR Kohn RR Luschin JH Apparent accelerated aging of human collagen in diabetes mellitus Diabetes 1975 24 902 904 10.2337/diabetes.24.10.902 170154

[B6] Fogarty AW Jones S Britton JR Lewis SA McKeever TM Systemic inflammation and decline in lung function in a general population: a prospective study Thorax 2007 62 515 520 10.1136/thx.2006.066969 17251312 PMC2117221

[B7] Hankinson JL Bang KM Acceptability and reproducibility criteria of the American Thoracic Society as observed in a sample of the general population Am Rev Respir Dis 1991 143 516 521 10.1164/ajrccm/143.3.516 2001060

[B8] Gardner RM Hankinson JL West BJ Evaluating commercially available spirometers Am Rev Respir Dis 1980 121 73 82 7352715 10.1164/arrd.1980.121.1.73

[B9] Schnack C Festa A Schwarzmaier-D'Assié A Haber P Schernthaner G Pulmonary dysfunction in type 1 diabetes in relation to metabolic long-term control and to incipient diabetic nephropathy Nephron 1996 74 395 400 10.1159/000189342 8893162

[B10] Davis TM Knuiman M Kendall P Vu H Davis WA Reduced pulmonary function and its associations in type 2 diabetes: the Fremantle Diabetes Study Diabetes Res Clin Pract 2000 50 153 159 10960726 10.1016/s0168-8227(00)00166-2

[B11] Marvisi M Bartolini L del Borrello P Brianti M Marani G Guariglia A Cuomo A Pulmonary function in non-insulin-dependent diabetes mellitus Respiration 2001 68 268 272 10.1159/000050509 11416247

[B12] Cebral J Mut F Sforza D Löhner R Scrivano E Lylyk P Putman C Clinical Application of Image-Based CFD for Cerebral Aneurysms Int j numer method biomed eng 2011 27 977 992 10.1002/cnm.1373 21822465 PMC3150562

[B13] Yeh HC Punjabi NM Wang NY Pankow JS Duncan BB Cox CE Selvin E Brancati FL Cross-sectional and prospective study of lung function in adults with type 2 diabetes: the Atherosclerosis Risk in Communities (ARIC) study Diabetes Care 2008 31 741 746 10.2337/dc07-1464 18056886 PMC2773203

[B14] Molitch ME Steffes MW Cleary PA Nathan DM Baseline analysis of renal function in the Diabetes Control and Complications Trial. The Diabetes Control and Complications Trial Research Group [corrected] Kidney Int 1993 43 668 674 10.1038/ki.1993.96 8455366

[B15] Stephenson JM Fuller JH Viberti GC Sjolie AK Navalesi R Blood pressure, retinopathy and urinary albumin excretion in IDDM: the EURODIAB IDDM Complications Study Diabetologia 1995 38 599 603 10.1007/BF00400730 7489844

[B16] Chance WW Rhee C Yilmaz C Dane DM Pruneda ML Raskin P Hsia CC Diminished alveolar microvascular reserves in type 2 diabetes reflect systemic microangiopathy Diabetes Care 2008 31 1596 1601 10.2337/dc07-2323 18492945 PMC2494655

[B17] Girach A Manner D Porta M Diabetic microvascular complications: can patients at risk be identified? A review Int J Clin Pract 2006 60 1471 1483 10.1111/j.1742-1241.2006.01175.x 17073842

[B18] Gilmour IJ Barbosa JJ Effect of kidney dysfunction on lung volume in patients with diabetes Diabetes Care 1991 14 333 335 10.2337/diacare.14.4.333 2060436

[B19] Wanke T Formanek D Auinger M Popp W Zwick H Irsigler K Inspiratory muscle performance and pulmonary function changes in insulin-dependent diabetes mellitus Am Rev Respir Dis 1991 143 97 100 10.1164/ajrccm/143.1.97 1986691

[B20] Raff H Bruder ED Jankowski BM The effect of hypoxia on plasma leptin and insulin in newborn and juvenile rats Endocrine 1999 11 37 39 10.1385/ENDO:11:1:37 10668639

[B21] Watschinger B Brunner C Wagner A Schnack C Prager R Weissel M Burghuber OC Left ventricular diastolic impairment in type 1 diabetic patients with microalbuminuria Nephron 1993 63 145 151 10.1159/000187173 8450905

[B22] Girach A Vignati L Diabetic microvascular complications–can the presence of one predict the development of another? J Diabetes Complications 2006 20 228 237 10.1016/j.jdiacomp.2006.03.001 16798474

